# Effects of Graphene Oxide-Gold Nanoparticles Nanocomposite on Highly Sensitive Foot-and-Mouth Disease Virus Detection

**DOI:** 10.3390/nano10101921

**Published:** 2020-09-25

**Authors:** Jong-Won Kim, Myeongkun Kim, Kyung Kwan Lee, Kwang Hyo Chung, Chang-Soo Lee

**Affiliations:** 1 Bionanotechnology Research Center, Korea Research Institute of Bioscience & Biotechnology (KRIBB) 125 Gwahak-ro, Yuseong-gu, Daejeon 34141, Korea; kimjw@kribb.re.kr (J.-W.K.); kmkun8510@kribb.re.kr (M.K.); lkk@kribb.re.kr (K.K.L.); 2 Dignostics Platform Research Section, Electronics and Telecommunications Research Institute (ETRI) 218 Gajeong-ro, Yuseong-gu, Daejeon 34129, Korea; hyo@etri.re.kr; 3 Department of Life and Nanopharmaceutical Science, College of Pharmacy, Kyung Hee University, Seoul 02447, Korea; 4 Department of Biotechnology, University of Science & Technology (UST), Daejeon 34113, Korea

**Keywords:** polymerase chain reaction, foot-and-mouth disease virus, graphene oxide, gold nanoparticle

## Abstract

The polymerase chain reaction (PCR) has become a powerful molecular diagnostic technique over the past few decades, but remains somewhat impaired due to low specificity, poor sensitivity, and false positive results. Metal and carbon nanomaterials, quantum dots, and metal oxides, can improve the quality and productivity of PCR assays. Here, we describe the ability of PCR assisted with nanomaterials (nano-PCR) comprising a nanocomposite of graphene oxide (GO) and gold nanoparticles (AuNPs) for sensitive detection of the foot-and-mouth disease virus (FMDV). Graphene oxide and AuNPs have been widely applied as biomedical materials for diagnosis, therapy, and drug delivery due to their unique chemical and physical properties. Foot-and-mouth disease (FMD) is highly contagious and fatal for cloven-hoofed animals including pigs, and it can thus seriously damage the swine industry. Therefore, a highly sensitive, specific, and practical method is needed to detect FMDV. The detection limit of real-time PCR improved by ~1000 fold when assisted by GO-AuNPs. We also designed a system of detecting serotypes in a single assay based on melting temperatures. Our sensitive and specific nano-PCR system can be applied to diagnose early FMDV infection, and thus may prove to be useful for clinical and biomedical applications.

## 1. Introduction

Foot-and-mouth disease (FMD) is a highly contagious and fatal disease of wild and domestic cloven-hoofed animals such as cattle, sheep, goat, and swine. It is caused by infection with the foot-and-mouth disease virus (FMDV). This RNA virus belongs to the genus *Aphthovirus* of the family of a *Picornaviridae* [1], and has been classified as serotypes O, A, C, SAT 1, SAT 2, SAT 3, and Asia 1 [2]. Since it can disseminate over long distances, FMD can severely restrict international trade in animals and related materials, and trigger massive economic damage because FMDV is highly infectious and can cause acute epidemics in FMD-free areas [3]. Various in vitro diagnostic methods such as enzyme-linked immunosorbent assays (ELISA) [4], hybridization assays [5], and polymerase chain reaction (PCR) [6,7,8,9,10,11,12,13], have been developed to detect various viruses. Molecular detection of FMDV using PCR and loop-mediated isothermal amplification (LAMP) is considered highly sensitive [14,15,16]. The sensitivity of PCR is high due to amplification of the target DNA and it a powerful molecular diagnostic technique. However, the utility of PCR is limited due to low specificity and efficiency due to time-consuming and temperature-dependent denaturation, annealing, and elongation steps that frequently generate false positive results. Thus, a highly sensitive, specific, and rapid method of detecting FMDV is required. Nanomaterial-assisted PCR (nano-PCR) has recently been developed to overcome these limitations and improve efficiency over conventional PCR [17,18,19]. Nanomaterials, including gold and magnetic nanoparticles, titanium oxide, quantum dots, and carbon-based materials have been used to develop PCR assays with outstanding sensitivity, specificity, and efficiency. These nanomaterials are thought to improve PCR efficiency and specificity via increased thermal conductivity and interaction with PCR reagents [20,21,22,23]. In nano-PCR, enhanced efficiency within shorter overall thermal cycling periods has been observed compared with conventional PCR due to simple addition of gold nanoparticles to PCR reaction mixtures [24,25,26,27].

We developed a nano-PCR system for the sensitive and specific detection of FMDV by enhancing PCR with graphene oxide-gold nanoparticles nanocomposite (GO-AuNPs) that supported DNA amplification and improved PCR efficiency by interacting with PCR reagents. We designed specific serotype O-, A-, and pan-type specific primers for real-time PCR with GO-AuNPs to distinguish between serotypes that have emerged in South Korea over several decades. The sensitivity of our nano-PCR system was 1000-fold higher than that of conventional PCR, and the enhanced efficiency could be precisely controlled by changing the GO-AuNPs content. This remarkable amplification ability would be useful for many critical circumstances, such as diagnosis of the early stages of various infectious diseases.

## 2. Materials and Methods

### 2.1. Materials

Graphene oxide in solution was purchased from Graphene Supermarket (Calverton, NY, USA). Gold (III) chloride trihydrate (HAuCl_4_·3H_2_O) and trisodium citrate (Na_3_C_6_H_5_O_7_) were from Sigma-Aldrich (St. Louis, MO, USA). The nPfu-forte polymerase was obtained from Enzymonics (Daejeon, Korea). Primers and FMDV genes were synthesized by Bioneer (Daejeon, Korea) and Integrated DNA technologies (IDT), respectively. SYBR Green I was purchased from Lonza (Rockland, ME, USA).

### 2.2. Preparation of GO-AuNPs Nanocomposite

The GO-AuNPs were prepared through a modification of the in situ reduction method [28,29]. Briefly, 5 mL of graphene oxide (1.5 mg/mL) was sonicated for 5 min; then, HAuCl_4_·3H_2_O (0.1 mg/mL, 100 mL) was added, and the mixture was incubated under magnetic stirring for 30 min to allow gold ions to adsorb onto graphene oxide surfaces. The solution was heated at 90 °C, and then the reducing agent, sodium citrate (0.035 g/mL, 1 mL), was added. The reaction was continued for 1 h, and the resultant solution was cooled to room temperature and washed three times by centrifugation at 3940× *g* with distilled water to remove free gold nanoparticles.

### 2.3. Characterization of GO-AuNPs

The GO-AuNPs were characterized by UV/vis spectroscopy, transmission electron microscopy (TEM), and zeta potential. The optical spectrum of GO-AuNPs was determined using a UV/Vis spectrophotometer (Optizen pop, Mecasys, Korea). Morphology and zeta potentials were assessed using a Talos F200X field-emission transmission electron microscope (TEM) (FEI Co., Hillsboro, OR, USA) at 200 kV, and dynamic light scattering (DLS; Zetasizer Nano ZS, Malvern Instruments, Malvern, UK), respectively.

### 2.4. Construction of FMDV Gene Plasmid

Serotype O- and A-type FMDV genes that originated from O-type (O/Andong/KOR/2010) and A-type (A/Pocheon/001/KOR/2010) strains were designed based on part of the RNA-dependent RNA polymerase sequence. The synthesized O- and A-type genes were cloned into pET-21a plasmids (Novagen, Inc., Madison, WI, USA). The constructed plasmids were transformed into the Escherichia coli (*E. coli*) host strain DH5α (RBC Bioscience Corp., New Taipei City, Taiwan). A single colony was selected and incubated in Luria-Bertani (LB) medium containing ampicillin (100 µg/mL) at 37 °C overnight. Cultured cells were harvested, and plasmids were prepared using extraction kits (Bioneer, Daejeon, Korea).

### 2.5. Real-Time PCR

We designed serotype O-, A-, and pan-type primers to investigate the performance of GO-AuNPs in real-time PCR (Table 1). The PCR mixture (total volume, 20 µL) consisted of 1X nPfu-forte buffer containing 200 µM each of dNTP, 1X SYBR Green I dye, 250 nM of each primer, 0.5 mg/mL bovine serum albumin (BSA), 0.5 U nPfu-forte polymerase, DNA templates, and optimal concentrations of AuNPs, GO, and GO-AuNPs. All real-time PCR reactions were performed on the CFX96 real-time PCR system (Bio-Rad Laboratories, Inc., Hercules CA, USA) and DNA was amplified using 40 cycles of denaturation at 95 °C for 10 s, annealing at 60 °C for 5 s, and extension at 72 °C for 20 s. We analyzed melting curves as fluorescent signals over a temperature range of 65 °C to 95 °C, that increased in 0.5 °C increments for 5 s after DNA amplification. The results were analyzed using CFX Maestro software (CFX96; Bio-Rad Laboratories, Inc., Hercules, CA, USA). Amplicons were loaded onto 2% agarose gels with a nucleic acid stain and resolved by electrophoresis at 100 V for 30 min. Bands were visualized using a UV lamp at a wavelength of 254 nm.

## 3. Results

### 3.1. Characterization of GO-AuNPs Nanocomposite

We evaluated the detection efficiency of FMDV by PCR using AuNPs, GO and GO-AuNPs, respectively. The GO-AuNPs were synthesized using a modification of in situ reduction [28,29], comprising seed-mediated growth on GO sheets, with three sizes of citrate-capped AuNPs (~10, 15, and 28 nm). Among them, GO-AuNPs with 15-nm AuNPs on GO sheets show the most efficient PCR performance (data not shown), thus, demonstrating that the size of AuNPs has a decisive effect on nano-PCR.

**Figure 1:**
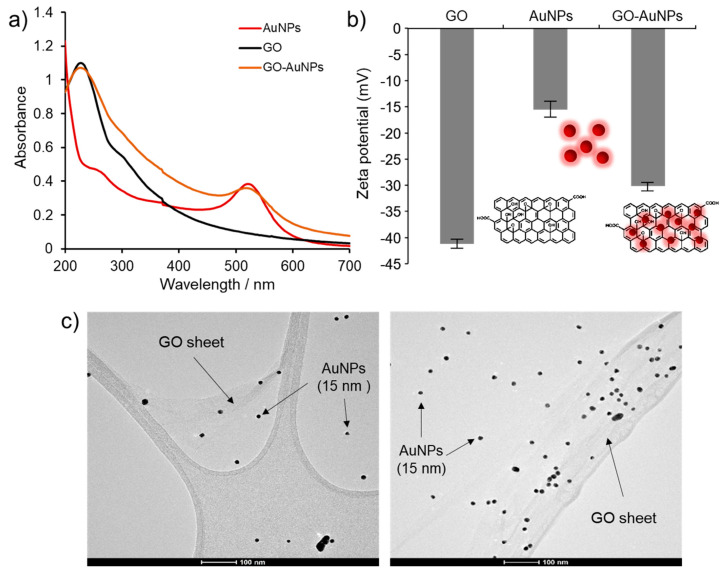
Characterization of GO-AuNPs. UV/Vis absorption spectra (**a**), zeta potentials (**b**) of AuNPs, GO, and GO-AuNPs, and TEM image (**c**) of GO-AuNPs.

We characterized the GO-AuNPs using UV/Vis spectroscopy, zeta potentials, and TEM (Figure 1). The optical properties of the nanomaterials were verified based on UV/Vis absorption spectra. Figure 1a shows an absorption peak of AuNPs and GO-AuNPs at 520 nm, which is typical of the localized surface plasmon resonance (LSPR) spectrum. The spectrum of GO and GO-AuNPs peaked at 288 nm, thereby indicating π-π* transitions for the C=C bonds and n-π* transitions for the C=O bonds of GO [28,30]. These results confirmed that GO-AuNPs had the optical characteristics of both AuNPs and GO, thereby implying successful absorption of AuNPs onto the surface of the GO sheets. We next evaluated the zeta potentials of GO-AuNPs to determine surface charges on the nanomaterials (Figure 1b). The estimated zeta potentials of AuNPs, GO, and GO-AuNPs were −15.43 ± 2.73, −41.87 ± 0.87, and −30.53 ± 1.36 mV, respectively. The average zeta potential of GO-AuNPs was less negatively charged (−30.53 ± 1.36 mV) than GO (−41.87 ± 0.87 mV), due to the coverage of AuNPs prepared from in situ reduction on the GO surface [31]. The size, shape, and distribution of GO-AuNPs examined by TEM showed that the spherical AuNPs were randomly distributed and localized on the surface of GO sheets with a diameter of 15.6 ± 1.3 nm, and morphological deformation was not evident (Figure 1c). These results showed that GO could serve as a supporting substrate for the hybrid material with characteristics of both GO and AuNPs. They also confirmed that GO could prevent self-aggregation by acting as a building block, while preserving good water dispersibility.

### 3.2. Nano-PCR with GO-AuNPs Nanocomposite

**Figure 2:**
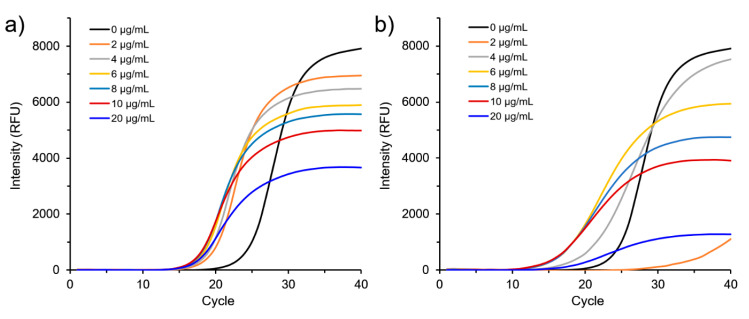
Effect of GO-AuNPs concentrations on nano-PCR using pan-type primers. Quantitation of FMDV O-type (**a**) and FMDV A-type (**b**) gene amplification.

The GO-AuNPs, including ~15-nm AuNPs immobilized onto the surface of GO sheets, were included as a component of the nano-PCR reaction. To investigate whether GO-AuNPs could enhance PCR detection of FMDV, we compared DNA amplification ability between GO-AuNPs-based, and conventional real time-PCR. The optimal conditions for nano-PCR were determined by varying GO-AuNPs concentrations and AuNP sizes on the GO sheets. We designed FMDV pan-type specific primers and applied these to real-time PCR with GO-AuNPs. Optimal concentrations were determined by including 2–20 µg/mL of GO-AuNPs. The optimal concentration of GO-AuNPs was ~10 µg/mL (Figure 2). Notably, 20 µg/mL of GO-AuNPs caused an appreciable decrease in signals. Thus, the concentration of AuNPs is crucial for enhancing PCR efficiency. GO-AuNPs can selectively bind to single-stranded DNA (ssDNA) through electrostatic interaction [23,32]. This improved PCR efficiency by functioning like a single-stranded DNA binding protein (SSB) that facilitates interactions between primers and templates during DNA replication in vivo. Therefore, adding >10 µg/mL of GO-AuNPs impeded the PCR reaction instead of facilitating it. Despite having low affinity, excess GO-AuNPs bound not only to ssDNA, but also to double-stranded DNA (dsDNA). Therefore, 10 µg/mL was again the optimal GO-AuNPs concentration for nano-PCR. Thus, we included this concentration in subsequent nano-PCR experiments.

We compared the performance of pristine AuNPs (0.2–2 nM) with GO (2–20 µg/mL) using FMDV O-type genes. The optimal concentrations were 0.8 nM and 4 µg/mL, respectively (Figure S1).

**Figure 3:**
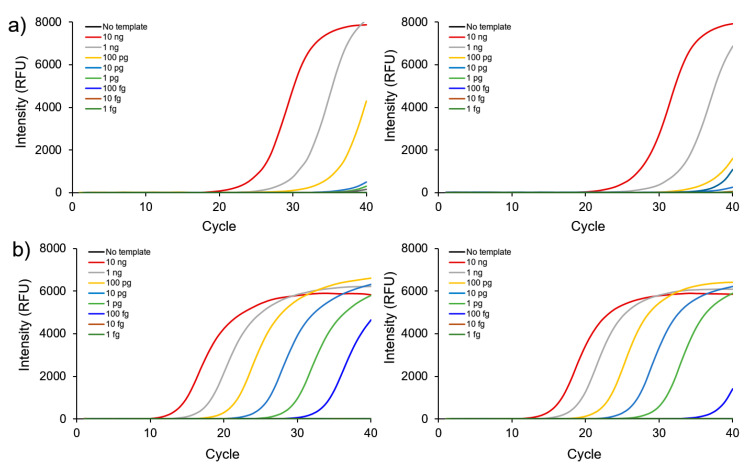
Effect of FMDV gene concentrations on nano-PCR using pan-type primers. Quantitation of FMDV gene amplification without (**a**) and with (**b**) GO-AuNPs. Left and right panels, O- and A-type genes, respectively.

**Figure 4:**
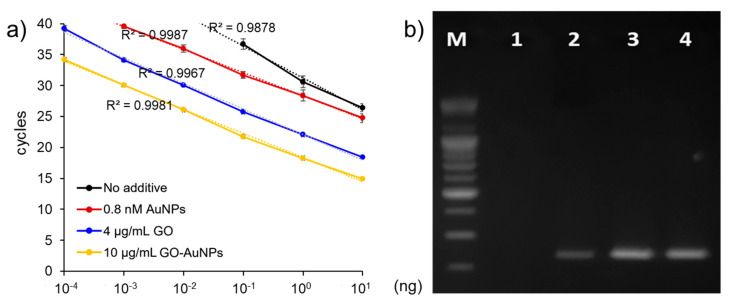
Comparison of additive efficiency in nano-PCR. (**a**) Standard curve of cycle values (Cq). (**b**) Size of nano-PCR product determined by agarose gel electrophoresis. Lane 1, no additives; Lanes 2–4, 0.8 nM AuNPs, 4 µg/mL GO, 10 µg/mL GO-AuNPs, respectively.

We next determined the sensitivity of detecting FMDV genes. Figure 3 shows consistent signals plotted according to increasing DNA template concentrations. The estimated detection limits of FMDV O- and A-type genes were 100 fg with and 100 pg without GO-AuNPs. These results indicated that the sensitivity of real-time PCR for FMDV detection was increased by ~1000 fold by adding GO-AuNPs to the PCR mixture. We directly compared the efficiency of GO-AuNPs with other nanomaterials using pristine AuNPs and GO as controls. The cycle quantitation values (Cq) of nano-PCR using AuNPs, GO, and GO-AuNPs were plotted. The standard curves in Figure 4a show a linear relationship from 100 fg to 10 ng for GO and GO-AuNPs. The results of no additives and of added AuNPs ranged from 100 pg to 10 ng and from 1 pg to 10 ng, respectively. That is, while the detection limits of GO-AuNPs and GO were in the order of 100 fg, those without additives and with AuNPs were in the order of 100 pg and 1 pg, respectively. Besides, GO-AuNPs had better amplification effects than GO in terms of the cycle quantitation (Cq) values at all points. Table 2 shows that the Cq values of GO-AuNPs were 4–5 cycles lower than those of GO. These findings confirmed that the effect of GO-AuNPs improved PCR performance by facilitating the powerful interactions between GO-AuNPs and PCR reagents due to the synergistic effect of GO and AuNPs [33]. Based on these results, we further confirmed the efficiency of each material by agarose gel electrophoresis (Figure 4b). Signals from bands of the PCR amplified products with GO and GO-AuNPs were more intense than those from the bands of PCR products with and without AuNPs. These results suggested that GO-AuNPs comprised the most effective enhancement material for detection of FMDV using nano-PCR.

### 3.3. Type-Specific Detection

Various antigens have been detected using lateral flow assays (LFA), which have also been applied in kits as rapid point-of-care tests (POCT). Two or more serotype-specific antibodies are immobilized on multiple lines (test and control lines) in one strip. However, FMDV has seven serotypes, among which serotypes O and A have recently spread worldwide, and especially in South Korea [34]. From this viewpoint, we designed a single reaction strategy to distinguish between serotypes. We also designed FMDV O- and A-type specific primers to produce different sizes of products after GO-AuNPs-based nano-PCR for FMDV type-specific detection. The specificity of nano-PCR with GO-AuNPs was evaluated using lambda DNA and *E. coli* genomic DNA as a control for optimization.

**Figure 5:**
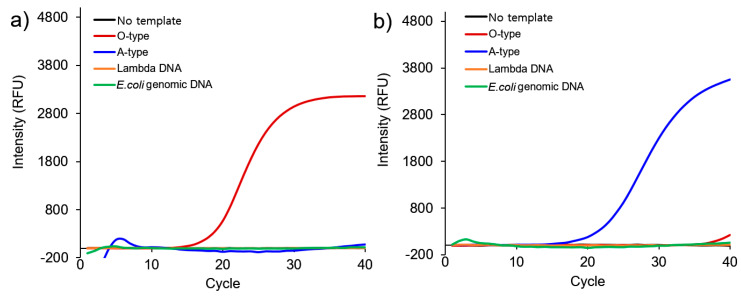
Specificity of nano-PCR with GO-AuNPs using O- and A-type specific primers and 10 ng of DNA templates. (**a**) O- and (**b**) A type-specific primers, respectively.

Figure 5 shows that both the O- and A-type specific primers worked specifically without amplifying other DNA templates. We also confirmed specific amplification using pan-type primers (Figure S2). We then prepared a mixture of O- and A-type specific primers to determine serotypes in a single reaction.

**Figure 6:**
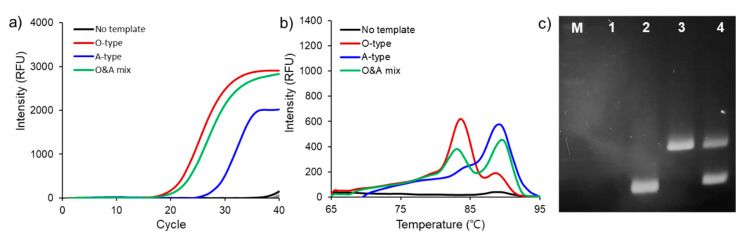
Real-time PCR of mixtures of O- and A-type specific primers and 10 ng of DNA templates. (**a**) Quantitation of FMDV gene amplification. (**b**) Results of melting curve derivatives. (**c**) Size of nano-PCR products determined by agarose gel electrophoresis. Lane: 1, no template; lane 2, O-type genes; lane 3, A-type genes; lane 4, mixture of O- and A-type genes.

Figure 6a shows fluorescent signals generated with O-, A-type genes and O- and A-type gene primers during nano-PCR. The types were distinguished by analyzing melting curves. The estimated melting temperatures (Tm) of O- and A-type gene products were 83.5 °C and 89.0 °C, respectively (Figure 6b). Furthermore, melting curves of O- and A-type gene mixture also showed two temperature peaks representing O- and A-types. Finally, the size of products after GO-AuNPs-based nano-PCR using the primer mixture was confirmed by agarose gel electrophoresis (Figure 6c). The two bands in lane 4 that resolved at 117 and 208 base pair (bp) corresponded to the O- and A-type gene products, respectively. These results indicates that our primers with GO-AuNPs are highly sensitive and specific for detecting FMDV serotypes in a single nano-PCR reaction.

## 4. Discussion

The rapid, specific, and sensitive detection systems of FMDV have been available for years due to high transmissibility and lethality of the infection. The present main diagnostic methods of FMDV detection include RT-LAMP, RT-PCR, and LFA. The LFA is a rapid and simple immunoassay, but the sensitivity and specificity are low compared with molecular diagnostic methods. Conventional RT-LAMP and RT-PCR are widely used to base final decisions on the presence or absence of infection because amplification of the target DNA enables sensitive detection in only small amounts of specimens. However, specificity and fidelity are reduced by non-specific reactions and sample contamination, which has been a recurring issue in molecular diagnosis. Therefore, we aimed to overcome these limitations by applying nano-PCR with GO-AuNPs to reliably detect FMDV. Various nanomaterials have been applied to PCR to diagnose various diseases PCR system. The main mechanisms of enhancing PCR efficiency are selective binding to ssDNA and thermal conductivity [35]. The citrate in AuNPs can selectively bind to ssDNA rather than dsDNA via electrostatic attraction [23,32]. Additionally, GO can interact with ssDNA, primers, and polymerase through electrostatic and π–π stacking interactions [18,36]. Owing to the unique features of both AuNPs and GO, GO-AuNPs have been proven to remarkably enhance PCR [33]. Furthermore, the good thermal conductivity of AuNPs and GO results in rapid temperature changes and improved PCR efficiency [26,37]. Therefore, GO-AuNPs have enormous utility and potential in diagnosis of FMDV.

## 5. Conclusions

We aimed to diagnose FMDV with high sensitivity and specificity using nano-PCR with GO-AuNPs. After optimizing the GO-AuNPs concentration and the AuNP size on GO sheets, the detection limits of nano-PCR for FMDV were enhanced by ~1000 fold compared with PCR assays without GO-AuNPs. Based on this, we designed a strategy for serotype-specific detection in a single nano-PCR reaction and distinguished FMDV serotypes O and A by melting temperatures during real-time PCR. Among various nanomaterials that have been tested to enhance PCR efficiency, the performance GO-AuNPs in nano-PCR was superior to that of pristine AuNPs and GO. This efficient method of FMDV detection based on nano-PCR offers considerable potential for other DNA amplifications tools, such as RT-LAMP. Overall, this approach should be useful for the clinical diagnosis and monitoring of FMDV infection.

## Figures and Tables

**Table 1:** Primer sequences used in this study.

Type of FMDV Gene	Amplicon Size (bp)	Sequence
O-type	117	Forward: GCCTTGGAACTCATAGAGAAAAG Reverse: CAAAACATCGACGATGCGC
A-type	208	Forward: GTTTGCACGGTGTGCTGG Reverse: CTTTTCTCCATGAGCTCTAGAGC
Pan-type	225	Forward: TGAGGAGGTGTTTCGCACA Reverse: GTGTAAGTGTCCAGCTCAACTC

**Table 2:** Cycle quantitation values (Cq) compared between control and additives including AuNPs, GO, and GO-AuNPs.

	Cycle Quantification Value (C_q_)			
Concentration (ng)	No Additive	0.8 nM AuNPs	4 μg/mL GO	10 μg/mL GO-AuNPs
10^1^	26.43	24.74	18.43	14.92
10^0^	30.57	28.40	22.05	18.28
10^−1^	36.69	31.71	25.72	21.77
10^−2^		35.97	30.07	26.09
10^−3^		39.52	34.13	30.07
10^−4^			39.16	34.19

## Supplementary Materials

The following are available online at https://www.mdpi.com/2079-4991/10/10/1921/s1, Figure S1: The effect of concentration of GO and AuNPs on nano-PCR using pan-type primers. Quantification of amplification of FMDV O-type genes using (**a**) GO and (**b**) AuNPs., Figure S2: Specificity of nano-PCR with GO-AuNPs using pan-type primers. 10 ng of DNA templates used.
