# Supplementary material for: Effects of Graphene Oxide-Gold Nanoparticles Nanocomposite on Highly Sensitive Foot-and-Mouth Disease Virus Detection
Source: Nanomaterials (Basel). 2020 Sep 25;10(10):1921. doi: 10.3390/nano10101921 (PMC7601864; doi:10.3390/nano10101921)
Supplement: Supplementary file 1 [file nanomaterials-10-01921-s001.pdf]

nanomaterials

Supplementary Materials

Effects of Graphene Oxide-Gold Nanoparticles Nanocomposite on Highly Sensitive Foot-and-Mouth Disease Virus Detection

Jong-Won Kim [1,2], Myeongkun Kim [1,2], Kyung Kwan Lee [1,2,3], Kwang Hyo Chung [2], Chang-Soo Lee [1,2,4,*]

1 Bionanotechnology Research Center, Korea Research Institute of Bioscience & Biotechnology (KRIBB) 125 Gwahak-ro, Yuseong-gu, Daejeon 34141, South Korea; kimjw@kribb.re.kr (J.-W.K.); kmkun8510@kribb.re.kr (M.K.); lkk@kribb.re.kr (K.K.L.)
2 Dignostics Platform Research Section, Electronics and Telecommunications Research Institute (ETRI) 218 Gajeong-ro, Yuseong-gu, Daejeon 34129, South Korea; hyo@etri.re.kr (K.H.C.)
3 Department of Life and Nanopharmaceutical Science, College of Pharmacy, Kyung Hee University, Seoul 02447, South Korea
4 Department of Biotechnology, University of Science & Technology (UST), Daejeon 34113, South Korea
* Correspondence: cslee@kribb.re.kr

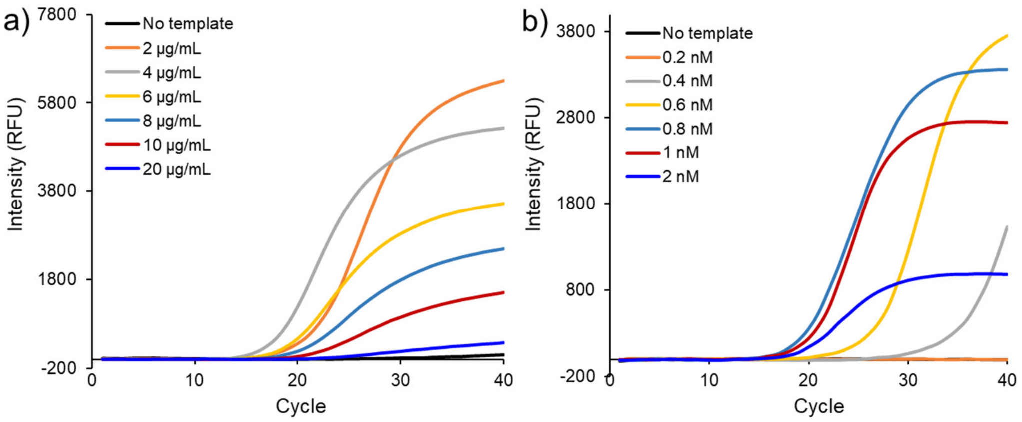

Figure S1. The effect of concentration of GO and AuNPs on nano-PCR using pan-type primers. Quantification of amplification of FMDV O-type genes using (**a**) GO and (**b**) AuNPs.

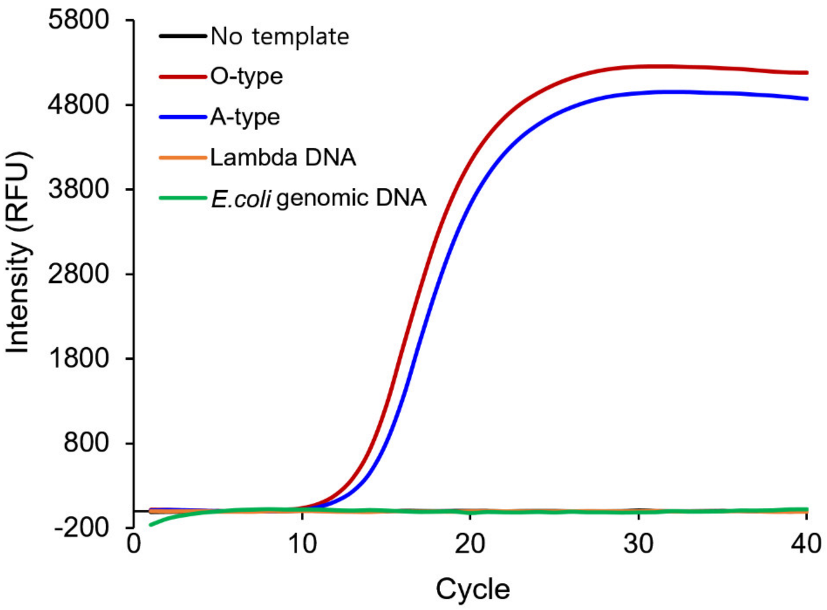

Figure S2. Specificity of nano-PCR with GO-AuNPs using pan-type primers. 10 ng of DNA templates used.

© 2020 by the authors. Licensee MDPI, Basel, Switzerland. This article is an open access article distributed under the terms and conditions of the Creative Commons Attribution (CC BY) license (http://creativecommons.org/licenses/by/4.0/).
